# High-Precision 2D-DOA Estimation Method for Millimeter-Wave Radar Based on Double-Parallel Linear Array and Joint IAA-RIT

**DOI:** 10.3390/s25082609

**Published:** 2025-04-20

**Authors:** Danyang Yu, Lei Du, Jie Bai, Yulin Chen

**Affiliations:** Division of Mechanics and Acoustics Metrology, National Institute of Metrology, Beijing 100029, China; yudanyang@nim.ac.cn (D.Y.); baijie@nim.ac.cn (J.B.); chenyl@nim.ac.cn (Y.C.)

**Keywords:** millimeter-wave radar, iterative adaptive approach, rotational invariance technique, double-parallel linear array, a few snapshots, signal coherence

## Abstract

High-precision two-dimensional direction of arrival (2D-DOA) estimation is an important mean of millimeter-wave radar for accurate target location. Aiming at the problems such as limited antenna aperture, signal coherence, and a few snapshots in millimeter-wave radar target detection, this paper proposes a 2D-DOA estimation method by using a joint iterative adaptive approach and rotational invariance technique (IAA-RIT) based on the double-parallel linear array. This method first constructs an iterative adaptive approach spectrum based on subarray 1 in the double-parallel linear array and then calculates the coupling angle estimate with the azimuth and the elevation. Secondly, based on the rotational invariance relationship between the two subarrays, the extended covariance matrices are respectively constructed, and the spatial smoothing technique is employed to decorrelate the signals. Then, the signal direction matrix is reconstructed based on the coupling angle estimate, and the rotational invariance relationship between the two subarrays is calculated to obtain another set of coupling angle estimates. Finally, the azimuth and the elevation are decoupled based on two sets of estimated coupling angles and the spatial geometry relation. Our experimental results show that IAA-RIT can estimate the coherent signal with high-precision 2D-DOA with a few snapshots and no additional angle matching.

## 1. Introduction

In recent years, millimeter-wave radar has been extensively utilized in the domains of intelligent vehicles and intelligent transportation, owing to its merits such as small volume, low cost, all-weather operation, and strong environmental adaptability [1,2]. The automotive millimeter-wave radar acquires the location of targets by measuring their distance, velocity, and angle. For the sake of the safety of autonomous driving [3], automotive millimeter-wave radar is required to obtain more precise road conditions and surrounding environment information [4,5]. High-resolution four-dimensional (distance-velocity-azimuth-elevation) imaging automotive millimeter-wave radar has emerged as a new research focus [6], and the demand for high-precision two-dimensional direction of arrival (2D-DOA) estimation of targets is on the rise.

Currently, to enhance the accuracy and resolution of the DOA estimation under the condition of a constrained array aperture, automotive millimeter-wave radars frequently employ the multiple-input and multiple-output (MIMO) system [7,8] by synthesizing a virtual equivalent array to expand the antenna array aperture. The most straightforward method for realizing the MIMO system is to utilize the time division multiplexing (TDM) technology [9]. The TDM technology achieves waveform orthogonality by having different transmitting antennas successively emit linear frequency modulation continuous waves (LFMCW), enabling easy channel separation at the receiving antenna [10], obtaining a larger equivalent virtual array and thereby achieving higher-precision DOA estimation.

Moreover, enhancing the resolution and accuracy of DOA estimation through signal processing algorithms is of great significance. Currently, numerous traditional DOA estimation algorithms [11,12] exist, such as the fast Fourier transform (FFT), conventional beam forming (CBF), and minimum variance distortionless response (MVDR). These methods are characterized by their simple principles and ease of implementation. However, due to the influence of the Rayleigh limit, their resolution poses challenges when applied to scenarios with high resolution requirements. In recent years, many scholars have proposed DOA super-resolution algorithms capable of breaking through the Rayleigh limit. The MUSIC and ESPRIT algorithms, which are based on subspace decomposition, utilize the orthogonality between the noise subspace and the target direction vector to acquire the target angle. Nevertheless, when dealing with coherent signals, the array aperture will be sacrificed to achieve accurate DOA estimation [13,14]. Given the spatial sparsity of radar echo signals, the compressed sensing theory can be applied to DOA estimation [15,16]. Examples include the orthogonal matching pursuit (OMP) and sparse Bayesian learning (SBL). Li et al. [17] proposed an OMP method with local optimization processing. This method reduces the computational complexity of the algorithm, yet its angular resolution requires further improvement. Zhang et al. [18] employed the expectation maximization algorithm to iteratively modify the discrete grid nodes of the sparse Bayesian model, thereby realizing an off-grid DOA estimation algorithm based on sparse Bayesian learning. Numerical experiments have verified that this algorithm can yield better DOA estimation results under low signal-to-noise ratio (SNR) and a few snapshots. However, its computational complexity remains relatively high.

The iterative adaptive approach (IAA) estimates the DOA with high precision by iteratively processing the signal covariance matrix and the energy matrix. It not only performs well under conditions of coherent signals and a few snapshots but also is applicable to arbitrary geometric arrays [19]. To address the issue of poor real-time performance of IAA, Xue et al. [20] utilized an appropriate Gohberg-Semencul type factorization to represent the covariance matrix and leveraged the Toeplitz structure of the matrix to reduce the computational cost of IAA. Chen et al. [21] proposed a Selective-IAA algorithm, which ignores non-regions of interest within the spatial domain and only considers DOA estimation within the regions of interest, thereby improving the real-time performance of the algorithm to some extent. Jie et al. [22] introduced a high-precision off-grid DOA estimation algorithm. This algorithm takes into account the mismatch between the actual target positions and the predefined discrete grid in practical problems, corrects the IAA power spectrum, calculates the power components and offsets after rough angle estimation, and performs alternating optimization to achieve high-precision DOA estimation.

In the case of millimeter-wave radar target detection, problems such as limited aperture of antenna array, signal coherence, and a few snapshots are often encountered. To solve these problems, this paper proposes a high-precision 2D-DOA estimation method that joints with an iterative adaptive approach and rotational invariance technique (IAA-RIT) based on double-parallel linear arrays. The rest of this paper is organized as follows. Firstly, the radar echo signal model based on a double-parallel array in TDM-MIMO mode is established in Section 2. Secondly, based on the radar data cube in Section 2, range-Doppler spectrum generation and Doppler phase compensation of the echo signal are carried out successively, and the algorithm principle of IAA-RIT is introduced in detail in Section 3. Thirdly, three sets of simulation experiments are set up to verify the effectiveness of the IAA-RIT algorithm in Section 4. Finally, the main content of this paper is summarized in Section 5.

## 2. Signal Model

**Figure 1:**
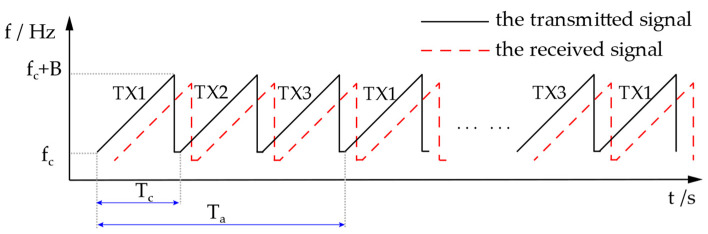
LFMCW signal waveform diagram under TDM-MIMO.

To enhance the array aperture and achieve superior angular resolution with a fixed number of array elements, a TDM-MIMO system is employed to generate a virtual equivalent array. Figure 1 illustrates the waveform diagram of the LFMCW signal within the TDM-MIMO system.

**Figure 2:**
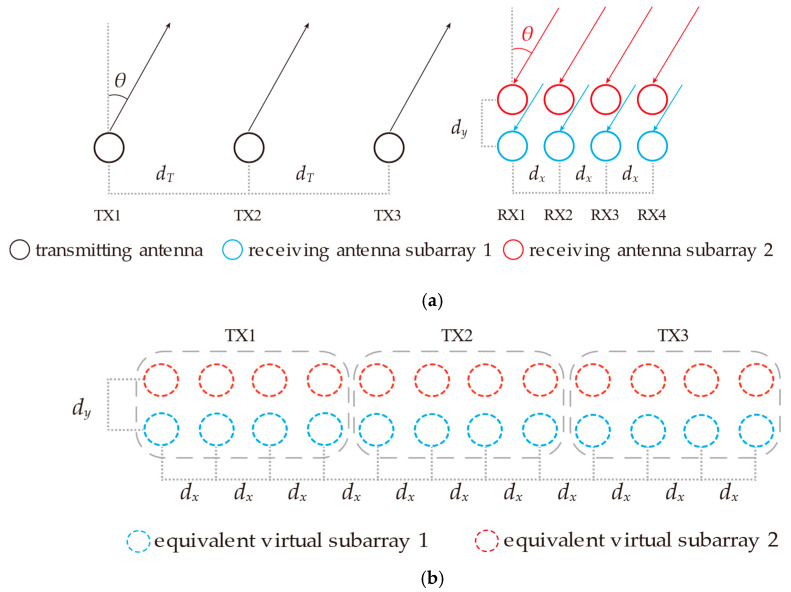
Schematic diagram of transmitting and receiving antenna and its virtual equivalent array: (**a**) Diagram of transceiver antenna array; (**b**) Diagram of equivalent virtual array.

Given the constraints imposed by the limited aperture of the antenna and the requirement for 2D-DOA estimation, this paper adopts a double-parallel linear array configuration. Compared to a uniform planar array, this configuration reduces the number of required array elements. Moreover, through the application of TDM technology, it is feasible to extend the effective array aperture while keeping the number of array elements constant. Figure 2 presents the schematic diagram of the antenna array alongside its equivalent virtual array.

Based on the aforementioned antenna array, assuming that the number of transmitting antennas is $N_{T}$ and the number of receiving antennas is $N_{R}$. Each transmitting antenna emits $N_{C}$ chirps in each frame, and each chirp contains $N_{S}$ sampling points with a sampling frequency of $F_{S}$. Supposing that the target moves at a constant and low velocity, and the range migration caused by the target movement is less than one range unit. Then, the discrete-time complex exponential signal received by the r-th receiving antenna of subarray 1 from the $n_{c}-\mathrm{th}$ chirp emitted by the t-th transmitting antenna can be represented by the following model:

(1) $$\begin{array}{cc} x_{t,r}^{1}(n_{c},n_{s}) & =s_{t,r}(n_{c},n_{s})+e_{t,r}(n_{c},n_{s})\\ & \approx \sum_{k=1}^{K}A_{k}e^{\phi_{k}}\exp[j2\pi (\frac{2R_{k}}{c}S+\frac{2v_{k}}{c}f_{c})\frac{n_{s}-1}{F_{S}}]\cdot \\ & \exp[j2\pi (n_{c}-1)T_{a}]\cdot \exp[j2\pi (t-1)\frac{2v_{k}}{c}f_{c}T_{c}]\\ & \exp[j2\pi (\frac{r+tN_{R}/2}{\lambda })d_{x}\cos\alpha_{k}]+e_{t,r}(n_{c},n_{s}) \end{array}$$

In Equation (1), $x_{t,r}^{1}(n_{c},n_{s})$ represents the received signal of subarray 1, $t=1,2,\ldots ,N_{T}$, $r=1,2,\ldots ,N_{R}$, $n_{c}=1,2,\ldots ,N_{C}$, where $N_{C}$ denotes the number of chirps within a frame, and $n_{s}=1,2,\ldots ,N_{S}$, where $N_{S}$ represents the number of sampling points for each chirp; $e_{t,r}(n_{c},n_{s})$ represents additive white Gaussian noise with a mean of 0 and a variance of $\sigma^{2}$; $A_{k}$ represents the complex amplitude of the signal sample; $\phi_{k}$ represents the fixed phase of the signal; $R_{k}$ and $v_{k}$, respectively, represent the radial distance and velocity of the k-th target; S represents the chirp rate of the linear frequency modulated continuous wave.

**Figure 3:**
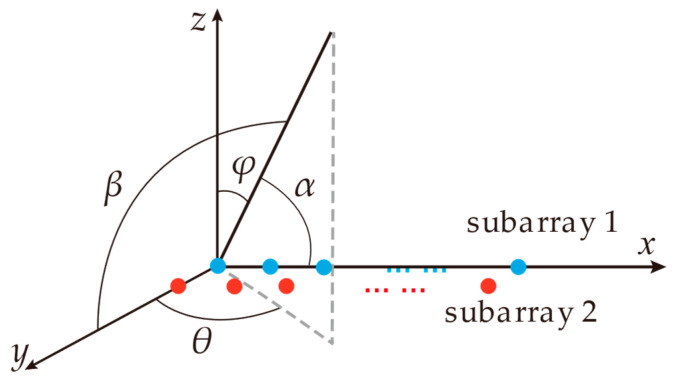
The geometric diagram between α and β with θ and φ.

The received signal $x_{t,r}^{2}(n_{c},n_{s})$ of subarray 2 exhibits a minor discrepancy compared to that of subarray 1, specifically in the fifth exponential term. The fifth exponential term of $x_{t,r}^{2}(n_{c},n_{s})$ can be expressed as $\exp\left\{j2\pi \left[\left(\frac{r+tN_{R}/2}{\lambda }\right)d_{x}\cos\alpha_{k}+d_{y}\cos\beta_{k}\right]\right\}$. The geometric relationship between α and β in the fifth exponential term with the azimuth θ and the elevation φ of the target is illustrated in Figure 3 as below, which can be expressed as

(2) $$\begin{array}{l} \cos\alpha =\sin\theta \sin\varphi \\ \cos\beta =\cos\theta \sin\varphi \end{array}$$

## 3. High-Precision 2D-DOA Estimation Method

### 3.1. Generation of the Range-Doppler Spectrum

Based on the sampled data as described in Equation (1), a radar data cube can be constructed, with its three dimensions corresponding to the ADC sampling number, chirp sequence number, and equivalent virtual antenna array size, respectively. A two-dimensional Fast Fourier Transform (2D-FFT) was applied to the raw echo data across the ADC and chirp dimensions for each receiving antenna, followed by non-coherent integration along the antenna dimension. Subsequently, constant false alarm rate (CFAR) processing and peak detection were performed on the resulting data to identify range-Doppler (RD) units containing targets. The RD domain data from these identified units were then separately extracted along the antenna dimension, with the data in each unit represented as

(3) $$\begin{array}{cc} X_{t,r}^{1} & =\sum_{m=1}^{M}A_{m}e^{j\phi_{m}}P_{N_{S}}(\frac{2\pi n_{s,p}}{N_{S}}-\omega_{R})P_{N_{C}}(\frac{2\pi n_{c,p}}{N_{C}}-\omega_{v})\\ & \cdot \exp[j2\pi (t-1)\frac{2v_{k}}{c}f_{c}T_{c}]\\ & \cdot \exp[j2\pi (\frac{r+tN_{R}/2}{\lambda })d_{x}\cos\alpha_{k}]+e_{t,r}(n_{c},n_{s}) \end{array}$$

In Equation (3), $X_{t,r}^{1}$ represents the data from a single peak unit extracted along subarray 1, where *M* denotes the number of targets within the extracted peak unit in the RD domain; $A_{m}$ represents the complex amplitude of the sampled signal for the $m_{th}$ target; $\phi_{m}$ represents a fixed phase; $n_{s,p}$ is the index of the distance dimension peak position, $0\leq n_{s,p}<N_{S}$; $n_{c,p}$ is the index of the Doppler peak location, $0\leq n_{c,p}<N_{C}$, and

(4) $$P_{N}\left(\omega \right)=\overset{N-1}{\underset{n=0}{\sum }}e^{-jn\omega }=\frac{\sin\left(\omega N/2\right)}{\sin\left(\omega /2\right)}e^{-j\omega \left(N-1\right)/2}$$

(5) $$\omega_{R}=\frac{2\pi }{F_{S}}(\frac{2v_{m}}{c}f_{c}+\frac{2R_{m}}{c}S)$$

(6) $$\omega_{v}=\frac{4\pi v_{m}T_{c}}{\lambda }$$

After undergoing identical processing, the data of subarray 2 exhibit only minor differences in the position of the third exponential term, which can be expressed as $\exp\left[j2\pi [(\frac{r+tN_{R}/2}{\lambda })d_{x}\cos\alpha_{k}+d_{y}\cos\beta_{k}]\right]$.

The RD spectrum, generated through 2D-FFT and processed using CFAR, effectively separates targets with varying distances and velocities. Subsequent processing of angular dimension information is confined to RD units that share the same distance and velocity parameters. This approach not only simplifies the DOA estimation process but also eliminates the need for additional pairing of angle, distance, and velocity. Furthermore, leveraging the known target velocities for phase compensation in angular dimension information enhances the accuracy of DOA estimation.

### 3.2. Doppler Phase Compensation Technique

When the MIMO transmitting antenna operates in TDM mode, an additional phase shift occurs due to target motion during the chirp duration. To achieve improved DOA estimation results, it is essential to conduct Doppler phase compensation on the angular dimension data in advance.

Equation (3) is converted to matrix form, which can be expressed as

(7) $$X_{0}^{1}=A_{0}s+e_{0}$$

(8) $$A_{0}=\left[a_{0}(\alpha_{1}),a_{0}(\alpha_{2}),\ldots ,a_{0}(\alpha_{m})\right]$$

(9) $$a_{0}(\alpha_{m})=a(\alpha_{m})⊕b$$

(10) $$a(\alpha_{m})={\left[1,e^{j\frac{2\pi }{\lambda }d_{x}\cos\alpha_{m}},\ldots ,e^{j\frac{2\pi }{\lambda }(N_{T}N_{R}-1)d_{x}\cos\alpha_{m}}\right]}^{T}$$

(11) $$b=\left[{\left[1,\ldots ,1\right]}_{N_{R}\times 1},\right.{\left[e^{j2\pi \frac{2v_{m}}{c}f_{c}T_{c}},\ldots ,e^{j2\pi \frac{2v_{m}}{c}f_{c}T_{c}}\right]}_{N_{R}\times 1},\ldots ,{\left.{\left[e^{j2\pi (N_{T}-1)\frac{2v_{m}}{c}f_{c}T_{c}},\ldots ,e^{j2\pi (N_{T}-1)\frac{2v_{m}}{c}f_{c}T_{c}}\right]}_{N_{R}\times 1}\right]}^{T}$$

(12) $$s={\left[s_{1},s_{2},\ldots ,s_{m}\right]}^{T}$$

where ⊕ represents the Hadamard product in Equation (9); Equation (11) is the extra phase shift caused by the moving target; Equation (12) represents the signal complex amplitude of m-th targets.

Define the Doppler compensation vector as

(13) $$\begin{array}{l} \;\tilde{b}=\left[{\left[1,\ldots ,1\right]}_{N_{R}\times 1},\right.{\left[e^{-j2\pi \frac{2{\tilde{v}}_{m}}{c}f_{c}T_{c}},\ldots ,e^{-j2\pi \frac{2{\tilde{v}}_{m}}{c}f_{c}T_{c}}\right]}_{N_{R}\times 1},\\ \ldots ,{\left.{\left[e^{-j2\pi (N_{T}-1)\frac{2{\tilde{v}}_{m}}{c}f_{c}T_{c}},\ldots ,e^{-j2\pi (N_{T}-1)\frac{2{\tilde{v}}_{m}}{c}f_{c}T_{c}}\right]}_{N_{R}\times 1}\right]}^{T} \end{array}$$

where ${\tilde{v}}_{m}$ represents the velocity estimate of the m-th target. Phase compensation is performed on the data in Equation (7), which can be expressed as

(14) $$X^{1}=X_{0}^{1}⊕\tilde{b}=\mathrm{As}+e$$

(15) $$A=\left[a(\alpha_{1}),a(\alpha_{2}),\ldots ,a(\alpha_{m})\right]$$

After the same processing, the data of subarray 2 are represented as the following matrix form

(16) $$X^{2}=A\Phi s+e$$

(17) $$\Phi =\mathrm{diag}(e^{j\frac{2\pi }{\lambda }d_{y}\cos\beta_{1}},e^{j\frac{2\pi }{\lambda }d_{y}\cos\beta_{2}},\ldots ,e^{j\frac{2\pi }{\lambda }d_{y}\cos\beta_{m}})$$

When $d_{y}$ is a constant, Φ represents the fixed phase offset between the two subarrays, that is, the inherent rotational invariance relationship between the two subarrays, which is called the rotational invariance factor. After the phase compensation of subarray 1 and subarray 2, the subsequent DOA estimation can be performed.

### 3.3. High-Precision 2D-DOA Estimation Method Combining IAA and RIT

To achieve high-precision 2D-DOA estimation under conditions of limited array aperture, signal coherence, and a few snapshots, a joint IAA-RIT method based on double-parallel linear arrays is proposed in this paper. This method effectively mitigates the impact of coherent signals and performs robustly with limited snapshots. Additionally, it eliminates the need for additional angle pairing, thereby reducing pairing errors. The joint IAA-RIT calculation process is given as follows.

Initially, the signal covariance matrix and energy matrix are iteratively updated via IAA by using subarray 1 from the double-parallel linear array. Once the iteration criteria are satisfied, spectral peaks are identified to estimate the coupled azimuth and elevation of the target ${\tilde{\alpha }}_{m}$.

Subsequently, an extended signal covariance matrix ***R*** is constructed using data from both subarray 1 and subarray 2. For processing coherent signals, spatial smoothing techniques are employed to decorrelate the signals.

Finally, the coupled angle estimates ${\tilde{\alpha }}_{m}$ are utilized to reconstruct the signal direction matrix $\tilde{A}$, $\tilde{A}$ and ***R*** are used to calculate the rotational invariance relationship between subarray 1 and subarray 2, yielding another set of coupled angles ${\tilde{\beta }}_{m}$. Equation (2) is then applied to decouple these angles, resulting in accurate estimates of the azimuth and elevation of the target. Prior to detailing the joint IAA-RIT method, the signal model defined by Equations (14) and (16) is reinterpreted as n-th snapshot data, which is given as follows

(18) $$X^{1}(n)=\mathrm{As}(n)+e$$

(19) $$X^{2}(n)=A\Phi s(n)+e$$

where n=1,2,…,N, and *N* indicates the total number of snapshots.

The following will be divided into three parts to introduce the specific content of the joint IAA-RIT.

In the first part, IAA is used to estimate DOA based on the data in subarray 1.

Firstly, defining the energy diagonal matrix ***P*** of the signal, the covariance matrix ***R*** of the signal, and the covariance matrix ***Q*** of the interference and noise outside the signal, respectively, which are given as follows

(20) $$\begin{array}{c} P_{m}=\frac{1}{N}\sum_{n=1}^{N}{\left|s_{m}\left(n\right)\right|}^{2} \end{array}$$

(21) $$\begin{array}{c} R=\frac{1}{N}X^{1}X^{1H}=A^{1}PA^{1H} \end{array}$$

(22) $$\begin{array}{c} Q\left(\alpha_{m}\right)=R-P_{m}a\left(\alpha_{m}\right)a^{H}\left(\alpha_{m}\right) \end{array}$$

where $P_{m}$ is the m-th diagonal element of ***P***. Defining the weighted least squares cost function as

(23) $$\begin{array}{c} \sum_{n=1}^{N}{\left\|X^{1}\left(n\right)-s_{m}\left(n\right)a\left(\alpha_{m}\right)\right\|}_{W}^{2} \end{array}$$

where ${\left\|x\right\|}_{W}^{2}=x^{H}Wx$. When the weighted matrix is $W=Q^{-1}\left(\theta_{k}\right)$, the estimation error of the least square estimator is the smallest. By the inverse lemma obtained from the matrix, ${\tilde{s}}_{m}\left(n\right)$ can be expressed as [19]

(24) $$\begin{array}{c} \begin{array}{c} {\tilde{s}}_{m}\left(n\right)=\frac{a^{H}\left(\alpha_{m}\right)R^{-1}X^{1}\left(n\right)}{a^{H}\left(\alpha_{m}\right)R^{-1}a\left(\alpha_{m}\right)} \end{array} \end{array}$$

Secondly, to compute the spatial power spectrum of the signal, it is essential to establish a predefined discrete grid dictionary for the spatial domain and set the number of grid points to *L (L >> M)*. Subsequently, the diagonal elements of the power matrix ***P*** are initialized, which can be formally defined as [19]

(25) $$\begin{array}{c} {\tilde{P}}_{l}=\frac{1}{{\left(a^{H}\left(\alpha_{l}\right)a\left(\alpha_{l}\right)\right)}^{2}N}\sum_{n=1}^{N}{\left|a^{H}\left(\alpha_{l}\right)X^{1}\left(n\right)\right|}^{2} \end{array}$$

where *l* = 1,2*,…,L.*

Finally, Equations (20), (21), and (24) are employed to iteratively update the covariance matrix ***R*** and the power matrix ***P*** until either the convergence condition is satisfied or the maximum number of iterations is reached. In this paper, the maximum number of iterations is set to 15 [19], and the convergence condition is defined as ${\left\|\mathrm{vec}\left(P^{\left(\iota +1\right)}-P^{\left(\iota \right)}\right)\right\|}_{2}/{\left\|\mathrm{vec}\left(P^{\left(\iota \right)}\right)\right\|}_{2}<\varepsilon$, where $\varepsilon =1\times 10^{-3}$ [23]. After the iteration process concludes, the coupling angle estimate ${\tilde{\alpha }}_{m}$ can be determined by identifying the spectral peak and utilizing the corresponding index value.

In the second part, the extended covariance matrix ***R*** is constructed by utilizing the data obtained from subarray 2 and subarray 1, as well as the rotational invariance relationship between them. Meanwhile, the signal direction matrix $\tilde{A}$ is reconstructed by using the results in the first part. Finally, the rotational invariance factor Φ containing the signal angle information is calculated. In this paper, this method is referred to as the Rotational Invariance Technique.

Specifically, the autocorrelation matrix of the received signal for subarray 1 is denoted as $R_{11}$, while the cross-correlation matrix between subarray 2 and subarray 1 is represented as $R_{21}$. Given the independence of the noise, both from itself and from the signal, these matrices can be, respectively, expressed as [24]

(26) $$\begin{array}{l} R_{11}=E[X^{1}X^{1H}]=\mathrm{AP}A^{H}+\sigma^{2}I\\ R_{21}=E[X^{2}X^{1H}]=A\Phi PA^{H} \end{array}$$

where $P=E[ss^{H}]$ is the signal covariance matrix; $\sigma^{2}$ is the variance of additive Gaussian white noise; ***I*** is the identity matrix.

Feature decomposition is performed on $R_{11}$, and $\varepsilon_{1},\varepsilon_{2},\ldots ,\varepsilon_{M}$ are set as the *M* larger eigenvalues of the matrix $R_{11}$. Under the assumption of white noise, the noise variance can be estimated from the average of $N_{T}N_{R}$-*M* smaller eigenvalues.

The following equation can be obtained by removing noise

(27) $$C_{11}=\mathrm{AP}A^{H}=R_{11}-\sigma^{2}I$$

Defining the extended covariance matrix $R\in {\mathbb{C}}^{N_{T}N_{R}\times N_{T}N_{R}}$, and

(28) $$R=R_{21}C_{11}^{+}$$

where $C_{11}^{+}=C_{11}^{H}{\left(C_{11}C_{11}^{H}\right)}^{-1}$.

If ***A*** and ***P*** are full-rank matrices, the diagonal elements in Φ are distinct, then the *M* non-zero eigenvalues of ***R*** are equivalent to the *M* diagonal elements of the matrix Φ. Furthermore, the eigenvectors corresponding to these eigenvalues are identical to the respective signal direction vectors [24].

(29) RA=AΦ

If coherent signals are present, the matrices $R_{11}$, $R_{21}$ and ***R*** can be reconstructed using the spatial smoothing technique in [25].

Subsequently, leveraging the angle estimate ${\tilde{\alpha }}_{m}$ derived from the IAA outcomes, the signal direction matrix $\tilde{A}$ is reconstructed by utilizing Equations (10) and (15).

(30) $$\tilde{A}=\left[a({\tilde{\alpha }}_{1}),a({\tilde{\alpha }}_{2}),\ldots ,a({\tilde{\alpha }}_{m})\right]$$

(31) $$a({\tilde{\alpha }}_{m})={\left[1,e^{j\frac{2\pi }{\lambda }d_{x}\cos{\tilde{\alpha }}_{m}},\ldots ,e^{j\frac{2\pi }{\lambda }(N_{T}N_{R}-1)d_{x}\cos{\tilde{\alpha }}_{m}}\right]}^{T}$$

Finally, the rotation invariant factor Φ is calculated.

(32) $$\Phi ={\tilde{A}}^{+}R\tilde{A}$$

where ${\tilde{A}}^{+}={\tilde{A}}^{H}{\left(\tilde{A}{\tilde{A}}^{H}\right)}^{-1}$. The coupling angle estimate ${\tilde{\beta }}_{m}$ can be obtained by using Equation (17).

In the third part, angle decoupling is conducted by utilizing coupling angle estimates ${\tilde{\alpha }}_{m}$, ${\tilde{\beta }}_{m}$ and their spatial geometric relationships with the azimuth and elevation.

Equation (2) is used to solve the azimuth $\theta_{m}$ and the elevation $\varphi_{m}$, which are expressed as follows

(33) $$\begin{array}{l} \theta_{m}=\arctan(\cos{\tilde{\alpha }}_{m}/\cos{\tilde{\beta }}_{m})\\ \varphi_{m}=\arcsin(\sqrt{\cos^{2}{\tilde{\alpha }}_{m}+\cos{\tilde{\beta }}_{m}}) \end{array}$$

The high-precision 2D-DOA estimation has been completed so far.

## 4. Simulation Experiment

To evaluate the performance of the DOA estimation method based on the IAA-RIT posed in this paper, three distinct sets of simulation experiments were designed, aiming to examine the resolution, accuracy, and capability of resolving coherent signals under varying SNR conditions and different snapshots. The simulations assumed a stationary radar system with targets moving at a constant low velocity and angle, and the antenna array was configured as a double parallel line array with three transmitters and eight receivers, as shown in Figure 2, where $d_{x}=d_{y}=d=\lambda /2$ and $d_{T}=4d=2\lambda$.

### 4.1. Verification Experiment on the Angular Resolution Performance of IAA-RIT

**Figure 4:**
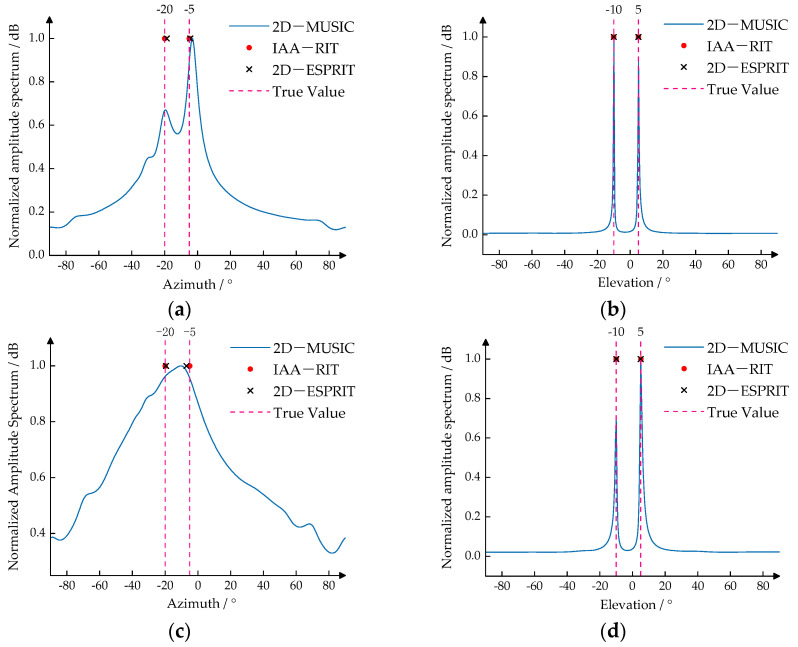
2D-DOA estimation results of different algorithms: (**a**) SNR = 25, *N* = 10, Azimuth; (**b**) SNR = 25, *N* = 10, Elevation; (**c**) SNR = 15, *N* = 10, Azimuth; (**d**) SNR = 15, *N* = 10, Elevation.

To evaluate the angle resolution capability of the IAA-RIT algorithm proposed in this paper under conditions of limited snapshots, simulation experiments were conducted using the IAA-RIT, 2D-MUSIC, and 2D-ESPRIT algorithms. The results of these experiments were then compared. In this experiment, the number of snapshots was set to *N* = 10, the SNRs for the two scenarios were set to 25 dB and 15 dB, and the target angles were configured as (−20°, 5°) and (−5°, −10°). The experimental outcomes are presented in Figure 4.

From the experimental results presented in Figure 4a,b, it is evident that when the SNR is 25 dB, all three algorithms can distinguish the two targets despite the relatively few snapshots. It can be observed from Figure 4c,d that when the SNR decreases to 10 dB, the 2D-MUSIC algorithm is unable to accurately distinguish the targets in the azimuth direction, and the 2D-ESPRIT algorithm also exhibits a relatively significant deviation when estimating the azimuth. Nevertheless, the IAA-RIT proposed in this paper demonstrates outstanding estimation performance in both the azimuth dimension and the elevation dimension. Not only can it accurately distinguish the targets, but also the calculated angles do not show obvious deviations either.

The experimental results presented in Figure 4 demonstrate that the IAA-RIT algorithm proposed in this paper achieves superior target resolution and angle estimation accuracy compared to the 2D-MUSIC and 2D-ESPRIT algorithms, particularly under conditions of limited snapshots and low SNR.

### 4.2. Verification of High-Precision Estimation Performance for the IAA-RIT

**Figure 5:**
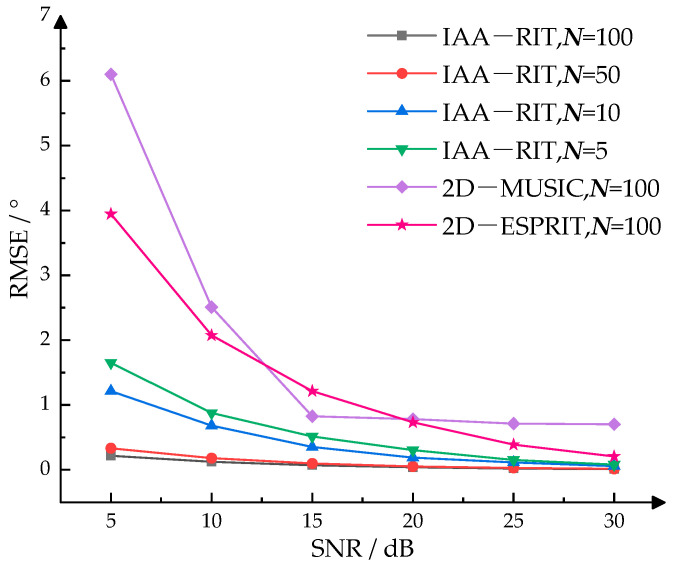
RMSE of IAA-RIT, 2D-MUSIC, 2D-ESPRIT at different SNR.

To evaluate the DOA estimation accuracy of the IAA-RIT algorithm proposed in this paper under conditions of limited snapshots, 200 Monte Carlo simulations were conducted. These simulations were performed using the IAA-RIT algorithm under varying conditions of snapshots and SNR. For comparison, 2D-MUSIC and 2D-ESPRIT algorithm simulations were also conducted under identical conditions. The target angles were set to (−20°, 5°) and (−5°, −10°). The experimental results were evaluated using the root mean square error (RMSE) as the metric for single-angle estimation accuracy, with the calculation formula provided in Equation (34). The detailed results are presented in Figure 5

(34) $$\begin{array}{c} RMSE=\sqrt{\frac{1}{2KN_{m}}\sum_{k=1}^{K}\sum_{i=1}^{N_{m}}{\left({\tilde{\theta }}_{i,k}-\theta_{k}\right)}^{2}+{\left({\tilde{\varphi }}_{i,k}-\varphi_{k}\right)}^{2}} \end{array}$$

where *K* is the number of targets; $N_{m}$ is the number of Monte Carlo experiments; ${\tilde{\theta }}_{i,k}$, ${\tilde{\varphi }}_{i,k}$ are, respectively, the azimuth estimates and the elevation estimates of the k-th target during the i-th Monte Carlo experiment; $\theta_{k}$, $\varphi_{k}$ are, respectively, the true value of the azimuth and the true value of the elevation of the k-th target.

According to the experimental results presented in Figure 5, the RMSE of the computational outcomes utilizing the IAA-RIT for 2D-DOA estimation is lower than that of the 2D-MUSIC and 2D-ESPRIT algorithms when the number of snapshots is less than 100. Specifically, the RMSEs obtained using IAA-RIT are 0.19098 and 0.30615 for 5 and 10 snapshots at an SNR of 20 dB, respectively. This indicates that the IAA-RIT can achieve high estimation accuracy even with a very limited number of snapshots under favorable SNR conditions. Furthermore, the RMSEs of the estimated angles using IAA-RIT are 0.3368 and 0.2184 for 50 and 100 snapshots at an SNR of 5 dB, respectively. These findings demonstrate that IAA-RIT can maintain good estimation accuracy under low SNR conditions when the number of snapshots is sufficiently large.

**Figure 6:**
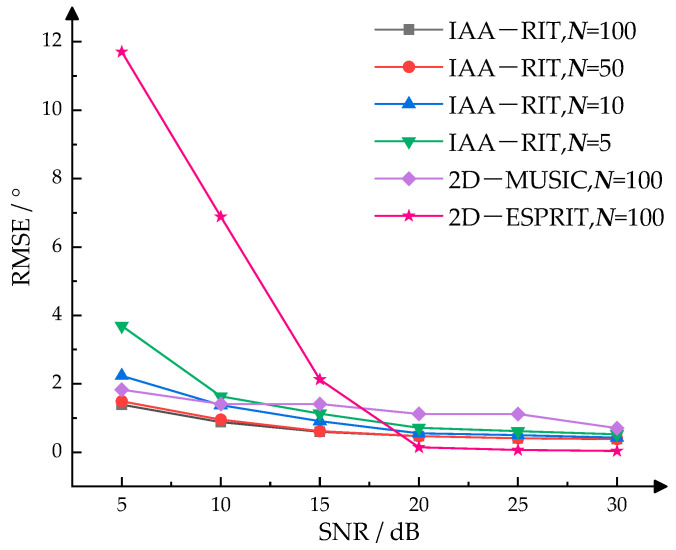
RMSE of IAA-RIT, 2D-MUSIC, 2D-ESPRIT in the angular edge region at different SNR.

In practical applications, the measurement range of azimuth angle for millimeter-wave radar typically spans from −60° to 60°, while that of elevation angle generally ranges from −20° to 20°. To evaluate the DOA estimation accuracy of the IAA-RIT proposed in this paper within the angular edge regions and under conditions of limited snapshots, 200 Monte Carlo experiments were executed. The IAA-RIT algorithm was employed under varying conditions of snapshots and different SNR. Additionally, simulation experiments using 2D-MUSIC and 2D-ESPRIT algorithms were carried out under identical conditions for comparison purposes. The target angles were configured as (−60°, 25°) and (70°, −20°). The experimental results were evaluated using the RMSE as the metric for single-angle estimation accuracy with the calculation formula provided in Equation (34). The detailed results are presented in Figure 6.

A comparison between the experimental results in Figure 5 and Figure 6 reveals that under identical conditions, the RMSE of 2D-DOA estimation using IAA-RIT is higher in the angular edge regions than in the angular central regions. As shown in Figure 6, although IAA-RIT exhibits weaker estimation capability for edge angles at low SNR and with limited snapshots, its RMSE remains slightly lower than that of 2D-MUSIC and 2D-ESPRIT. Specifically, the RMSE of IAA-RIT is 0.71413° with five snapshots and 0.46955° with 50 snapshots at an SNR of 20 dB. Similarly, increasing the number of snapshots reduces the RMSE of IAA-RIT at SNRs of 25 dB and 30 dB, consistently outperforming 2D-MUSIC. This indicates that IAA-RIT achieves satisfactory estimation accuracy under favorable SNR conditions, and its precision can be further improved by increasing snapshots. However, when SNR ≥ 20 dB, the RMSE of IAA-RIT across different snapshots remains higher than that of 2D-ESPRIT with 100 snapshots. Nevertheless, 2D-ESPRIT suffers from excessive errors at low SNR, demonstrating inferior robustness compared to IAA-RIT.

Overall, the experimental results in Figure 5 and Figure 6 confirm that the proposed IAA-RIT method exhibits robust 2D-DOA estimation performance under both low SNR and low snapshots conditions. The 2D-DOA estimation accuracy of IAA-RIT is lower in the angular edge regions than in the angular central regions, but its performance improves with higher SNR or an increased number of snapshots.

### 4.3. Verification Experiment on the Angle Resolution Performance and Estimation Accuracy of Coherent Source

**Figure 7:**
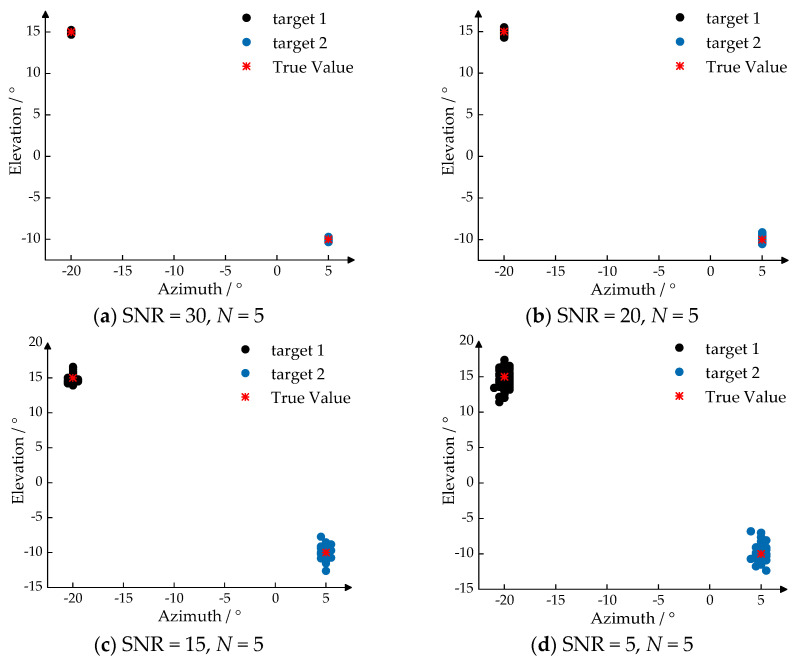
2D-DOA estimation results of the IAA-RIT algorithm with different SNR for a few snapshots of data and coherent sources.

To evaluate the 2D-DOA estimation performance of the IAA-RIT algorithm proposed in this paper under conditions of limited snapshots and source coherence, 200 Monte Carlo simulations were conducted. The simulations aimed to assess the angle estimation accuracy of the algorithm. The target angles were set at (−20°, 15°) and (5°, −10°). The experimental results are presented in Figure 7.

From the experimental results depicted in Figure 7, it is evident that the IAA-RIT can accurately estimate the angle of arrival for coherent signals with a minimal number of snapshots. As the SNR progressively increases, the target landing points from the Monte Carlo simulations become increasingly concentrated, indicating enhanced precision in 2D-DOA estimation using IAA-RIT even with limited snapshots. The experimental findings demonstrate that the IAA-RIT achieves high-performance 2D-DOA estimation under conditions of good SNR and low snapshots for coherent signals.

Through these three sets of experiments, it is clear that the IAA-RIT based on a double-parallel linear array possesses angle resolution capabilities even with limited snapshots, enabling high-precision 2D-DOA estimation of coherent signals when SNR conditions are favorable.

## 5. Conclusions

A 2D-DOA estimation method based on the joint IAA-RIT is proposed in this paper, which does not require additional angle matching. This method addresses the challenges of limited array aperture, signal coherence, and insufficient snapshots in automotive millimeter-wave radar target detection. The IAA-RIT algorithm utilizes a double-parallel linear array configuration. It constructs an IAA spectrum using subarray 1 to reconstruct the signal direction matrix and builds an extended covariance matrix through both submatrices. Consequently, it calculates the rotation-invariant relationship between the two submatrices and ultimately estimates the azimuth and the elevation of the targets via angle decoupling. Experimental results demonstrate that the 2D-DOA estimation performance of this algorithm surpasses that of 2D-MUSIC and 2D-ESPRIT algorithms under identical array configurations. At high SNR levels, the IAA-RIT can achieve high-precision 2D-DOA estimation with very few snapshots while ensuring algorithm effectiveness without additional angle matching, even in the presence of coherent signals.

For the following work, we will conduct a further study on the field verification method in actual traffic for the proposed 2D-DOA estimation algorithm based on the joint IAA-RIT by promoting the roadside reference facility established at the National Institute of Metrology, China [26] on the No. G92 motorway of China.

## Data Availability

The original contributions presented in this study are included in this article; further inquiries can be directed to the corresponding author.
